# A Novel Mutation in the *HSD17B10* Gene of a 10-Year-Old Boy with Refractory Epilepsy, Choreoathetosis and Learning Disability

**DOI:** 10.1371/journal.pone.0027348

**Published:** 2011-11-22

**Authors:** Laurie H. Seaver, Xue-Ying He, Keith Abe, Tina Cowan, Gregory M. Enns, Lawrence Sweetman, Manfred Philipp, Sansan Lee, Mazhar Malik, Song-Yu Yang

**Affiliations:** 1 Hawai'i Community Genetics, Kapi'olani Medical Specialists, Honolulu, Hawaii, United States of America; 2 Department of Pediatrics, John A. Burns School of Medicine, Honolulu, Hawaii, United States of America; 3 Department of Neurochemistry, New York State Institute for Basic Research in Developmental Disabilities, New York, New York, United States of America; 4 Department of Pathology, Stanford University School of Medicine, Stanford, California, United States of America; 5 Department of Pediatrics, Stanford University School of Medicine, Stanford, California, United States of America; 6 Institute of Metabolic Disease, Baylor Research Institute, Dallas, Texas, United States of America; 7 Department of Chemistry, Lehman College of the City University of New York, New York, New York, United States of America; Charité Universitätsmedizin Berlin, NeuroCure Clinical Research Center, Germany

## Abstract

Hydroxysteroid (17beta) dehydrogenase 10 (HSD10) is a mitochondrial multifunctional enzyme encoded by the *HSD17B10* gene. Missense mutations in this gene result in HSD10 deficiency, whereas a silent mutation results in mental retardation, X-linked, syndromic 10 (MRXS10). Here we report a novel missense mutation found in the *HSD17B10* gene, namely c.194T>C transition (rs104886492), brought about by the loss of two forked methyl groups of valine 65 in the HSD10 active site. The affected boy, who possesses mutant HSD10 (p.V65A), has a neurological syndrome with metabolic derangements, choreoathetosis, refractory epilepsy and learning disability. He has no history of acute decompensation or metabolic acidosis whereas his urine organic acid profile, showing elevated levels of 2-methyl-3-hydroxybutyrate and tiglylglycine, is characteristic of HSD10 deficiency. His HSD10 activity was much lower than the normal control level, with normal β-ketothiolase activity. The c.194T>C mutation in *HSD17B10* can be identified by the restriction fragment polymorphism analysis, thereby facilitating the screening of this novel mutation in individuals with intellectual disability of unknown etiology and their family members much easier. The patient's mother is an asymptomatic carrier, and has a mixed ancestry (Hawaiian, Japanese and Chinese). This demonstrates that HSD10 deficiency patients are not confined to a particular ethnicity although previously reported cases were either Spanish or German descendants.

## Introduction

The *HSD17B10* gene maps to Xp11.2 and encodes the enzyme hydroxysteroid (17beta) dehydrogenase 10 (HSD10) (see OMIM300256 at http://www.ncbi.nlm.nih.gov/entrez/dispomim.cgi?id=300256), which catalyzes the oxidation of steroids, fatty acids, and xenobiotics [1], [2]. In contrast to other types of hydroxysteroid (17beta) dehydrogenases, HSD10 is localized in mitochondria as a result of the non-cleavable targeting sequence at its N-terminal [3], [4]. This multifunctional enzyme is found in various brain regions [5], and its levels are significantly elevated in Alzheimer disease, Down syndrome, and multiple sclerosis [5], [6]. Duplication of the *HSD17B10* gene also promotes idiopathic mental retardation [7]. Furthermore, missense mutations in this gene result in an X-linked mental retardation, namely HSD10 deficiency [8] (OMIM#300438), formerly 2-methyl-3-hydroxybutyryl-CoA dehydrogenase deficiency [9].

Over the last decade, the phenotypic spectrum associated with HSD10 deficiency has expanded to include cases associated with early neonatal or infant death [10] and psychomotor retardation without regression [11], [12]. No case has been associated with episodic metabolic decompensation although severe lactic acidosis, reminiscent of mitochondrial disease, has been reported [10]. The complex neurologic phenotype reported to date has included developmental delay, hypotonia, dysarthria, ataxia, choreiform movement disorder, seizures (often reported to be myoclonic), and progressive loss of vision and/or hearing [10]–[14]. Hypertrophic cardiomyopathy is also reported [10], [11].

Results of magnetic resonance imaging (MRI) have also been variable but several authors have noted frontotemporal atrophy [10], [12], basal ganglia abnormality, and periventricular white matter disease [12]. Normal brain MRI has also been reported in infancy and childhood [14] and in one adult male [13].

A missense mutation in HSD10, namely p.R130C, has been detected in at least half of unrelated individuals, including one female with HSD10 deficiency [8]–[10]. Here we report a novel mutation identified in the *HSD17B10* gene responsible for a neurological syndrome in a 10-year-old boy.

## Results

### Case History

A 10-year-old boy of mixed ancestry (Portuguese, Hawaiian, Japanese, and Chinese) was evaluated for medically refractory epilepsy and previous diagnosis of pervasive developmental disorder (PDD). Birth history revealed that he was born appropriate for gestational age (AGA) at term to a 33-year-old gravida 2 para 2 mother. He appropriately met his early motor and language milestones. Developmental regression was noted at age 2–3 years. Gait became unsteady at age 3–4 years, associated with hyperkinetic involuntary movement disorder. Partial complex seizures lasting about 10–30 seconds developed at 3 to 4 years of age. Electroencephalography (EEG) at age 4 years showed diffuse background slowing and multifocal spike or polyspike activity without associated clinical seizures during the study. Treatment with carbamazepine and then subsequently oxcarbazepine seemed to lessen the frequency of seizures, but did not completely control them. At 6 to 7 years of age, seizures also included brief myoclonic jerks. EEG repeated at 7 years of age showed rare paroxysms of bifrontal high amplitude spike and slow wave discharges. Levetiracetam was added to the oxcarbazepine therapy for a period of time but was discontinued due to adverse effects at higher doses. Valproic acid was later started as an adjunct therapy.

**Figure 1:**
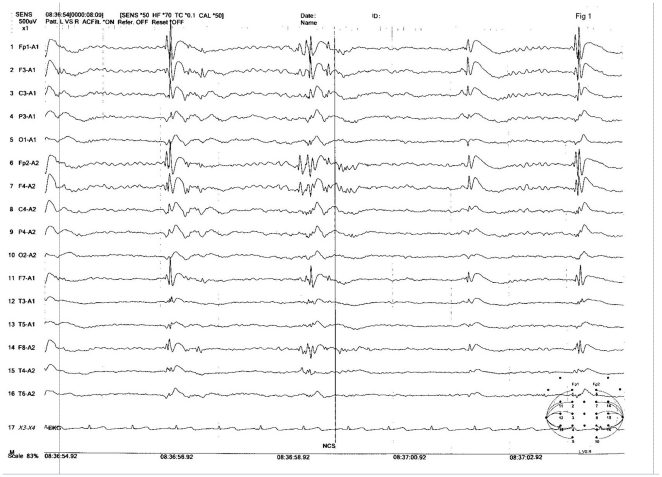
Electroencephalography of the affected boy at 10 years of age.

Over subsequent years, seizure activity and symptoms were variable, including brief absence seizure, myoclonic seizure, and other indeterminate seizure types including paroxysms of head nodding, eye rolling/gaze deviation, rocking of the trunk, and bilateral extremity posturing, all lasting on the order of 5 to 20 seconds in duration occurring from 2 to 5 times to up to 20 times per day. EEG repeated at 10 years of age showed predominantly generalized frequent very high amplitude spike-slow waves and polyspike-slow wave discharges during sedated sleep (Fig. 1). Due to findings of more convincing generalized discharges, oxcarbazepine was tapered off in favor of more broad spectrum antiseizure medication. He was most recently starting on lamotrigine in combination with valproic acid.

Based on the suspected diagnosis of 17-beta-hydroxysteroid dehydrogenase X (HSD10) deficiency (formerly 2-methyl-3-hydroxybutyryl-CoA dehydrogenase deficiency) treatment with mild protein restriction (1.5–2.0 g/kg/day) and oral carnitine was instituted. Compliance with diet has been difficult secondary to poor appetite and eating habits.

Past medical history is significant for one previous hospitalization for bronchiolitis during infancy. He has no history of acute decompensation or metabolic acidosis. He exhibits moderate cognitive impairment, repeated one year in school, is in a self-contained special education classroom setting, and receives speech, occupational and physical therapies.

Family history is negative for similarly affected individuals. He has one 17-year-old sister who is healthy and well. His nonconsanguinous parents are reportedly healthy. His mother completed two years of college and works in the medical field. He has one maternal aunt with two daughters, all reportedly healthy and without neurologic symptoms. His mother has a maternal half brother with Bell's palsy but no movement disorder or seizures. His maternal aunt and uncle both completed high school without need for educational assistance.

### Physical and Neurological Examination

When this boy was first evaluated at age 7 years, his height was at the 10^th^–25^th^ percentile, weight at the 10^th^ percentile, and head circumference <the 5^th^ percentile. Since then he has exhibited poor weight gain such that weight is now <the 3^rd^ percentile (BMI 13.1). He has no significant dysmorphic features or organomegaly. Ophthalmologic evaluation revealed bilateral optic nerve pallor and significant myopia.

His neurologic examination is significant for cognitive delay and mild impairment in social interactions. He has normal to mildly decreased muscle tone, normal deep tendon reflexes, down-going toes, dysarthria, ataxia, and near constant hyperkinetic choreoathetoid movements of the extremities and head. He does not exhibit nystagmus. Computerized tomography (CT) of the brain at age 3 ½ was normal. Magnetic resonance imaging (MRI) of the brain at age 7 years and 10 years were normal. Brain magnetic resonance spectroscopy (MRS) has not been approved by insurance.

### Biochemical Laboratory Evaluation

Normal data were obtained in the following tests: serum ammonia, creatine kinase (CK), electrolytes, liver transaminases, lipid profile (total cholesterol, triglycerides, LDL and HDL), serum lactic acid, complete blood count, anti-nuclear antibody (ANA) titer, anti-streptolysin O (ASO) titer, serum copper and ceruloplasmin, plasma amino acids, total and free carnitine, acylcarnitine/free carnitine ratio, acylcarnitine profile, plasma and urine creatine and guanidinoacetic acid and biotinidase activity. Qualitative urine organic acid screening revealed abnormal elevations of 2-methyl-3-hydroxybutyric acid and tiglyglycine, with no abnormal elevation of 2-methylacetoacetic acid. Serial quantitative urine organic acids examinations revealed persistent elevation of 2-methyl-3-hydroxybutyric acid and tiglylglycine (Table 1), again with no 2-methylacetoacetic acid detected. Beta-ketothiolase activity measured in skin fibroblasts was mildly low at 8.6 mU/mg protein (reference range 8.9–20.6) but its activity stimulated normally in the presence of potassium. Succinyl CoA-3-keto transferase (SCOT) activity, also measured in skin fibroblasts, was normal (4.1 mU/mg protein with reference range 2.6–8.6). The measurement of HSD10 (formerly MHBD) activity in lymphoblastoid cells of normal controls was 4.3±2.0 mU/mg protein (n = 4) similar to that previously reported 5.7±1.3 mU/mg protein [12], whereas the HSD10 activity measured in the patient's lymphoblastoid cells was decreased at 2.17 mU/mg protein. Mitochondrial electron transport chain enzymes measured in fibroblasts were normal (Table 2). Levels of organic acids (tiglylglycine and 2-methyl-3-hydroxybutyrate ) in urine of the patient's mother and sister were also determined, and found to be no greater than the normal adult level (tiglylglycine <8 and 2-methyl-3-hydroxybutyrate <10 mmole/mole creatine, respectively).

**Table 1:** Concentrations of urine organic acids (mmole/mole creatine) at different age.

Patient age:	2-Methyl-3-hydroxybutyric acid	Tiglylglycine
8 y 4 m	31	32
8 y 8 m	18	9
10 y 3 m	152	66
10 y 4 m	80	40
Normal children (age: 2 to 12-year-old)	<14	<6

**Table 2:** Activities of mitochondrial respiratory chain enzymes in fibroblasts.

Enzyme	*Specific activity in patient's cells*nmol/min/mg protein (% of mean)	*Normal ranges*nmol/min/mg protein
ETC Complex I	499 (83)	602+/−316 (276–806)
ETC Complex I+III (Total)Rotenone sensitive	249 (81)76.5 (76)	307+/−120 (170–410)100+/−40 (50–136)
ETC Complex II	5.84 (77)	7.6+/−5.9 (2.1–13.1)
ETC Complex II+III	13.4 (65)	20.6+/−11.7 (8.2–28.6)
ETC Complex IV	23.3 (89)	26.2+/−17.5 (8.2–42.4)
Citrate synthase (control)	13.9	(12.9–60.6)

### Molecular Genetic Study

**Figure 2:**
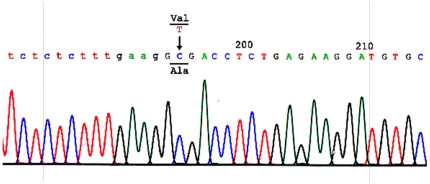
Mutation on the *HSD17B10* gene of patient with a clinical diagnosis of HSD10 deficiency. Chromatogram of the forward sequence of the *HSD17B10* gene from the patient showing c.194T>C transition. The nucleotide sequence of intron 2 is indicated by lower case. This mutation resulted in mutant HSD10(p.V65A).

**Figure 3:**
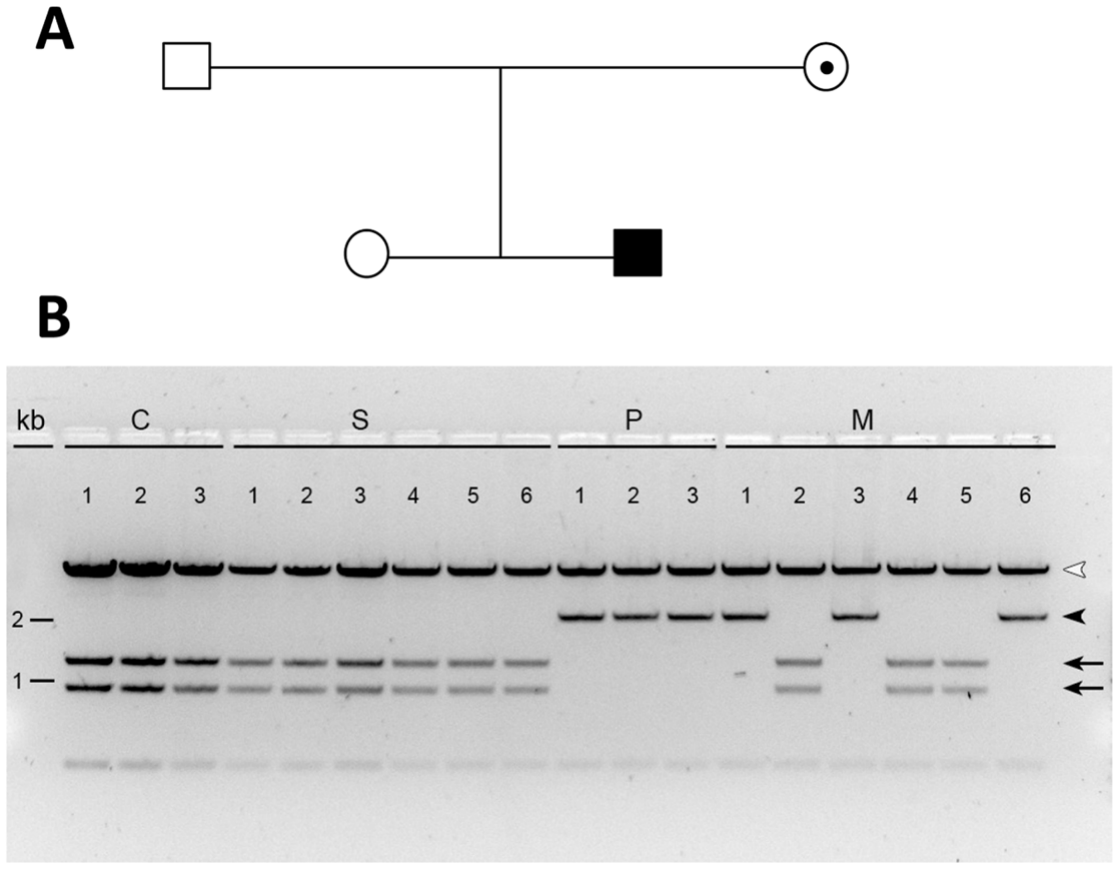
Detection of the c.194T>C variant in the *HSD17B10* gene by RFLP analysis. The pGEM-T Easy vectors harboring the *HSD17B10* gene cloned from genomic DNA of a normal control [**C1–3**] (lanes 1–3), the patient's sister [**S1–6**] (lanes 4–9), the patient [**P1–3**] (lanes10–12), and the patient's mother [**M1–6**] (lanes 13–18) were digested by *BstE*II and then separated on a 1% agarose gel. Amounts of DNA loaded were 1 mg on lanes 1 and 2, 0.75 mg on lanes 3 and 6, and 0.5 mg on all the other lanes. A 2.2 kb fragment (indicated by an arrowhead) results from an allele carrying this variant. For a wild-type allele, this fragment is chopped into two shorter fragments (1.3 kb and 0.9 kb) as indicated by arrows. The vector is in the largest band indicated by an empty arrowhead.

**Figure 4:**
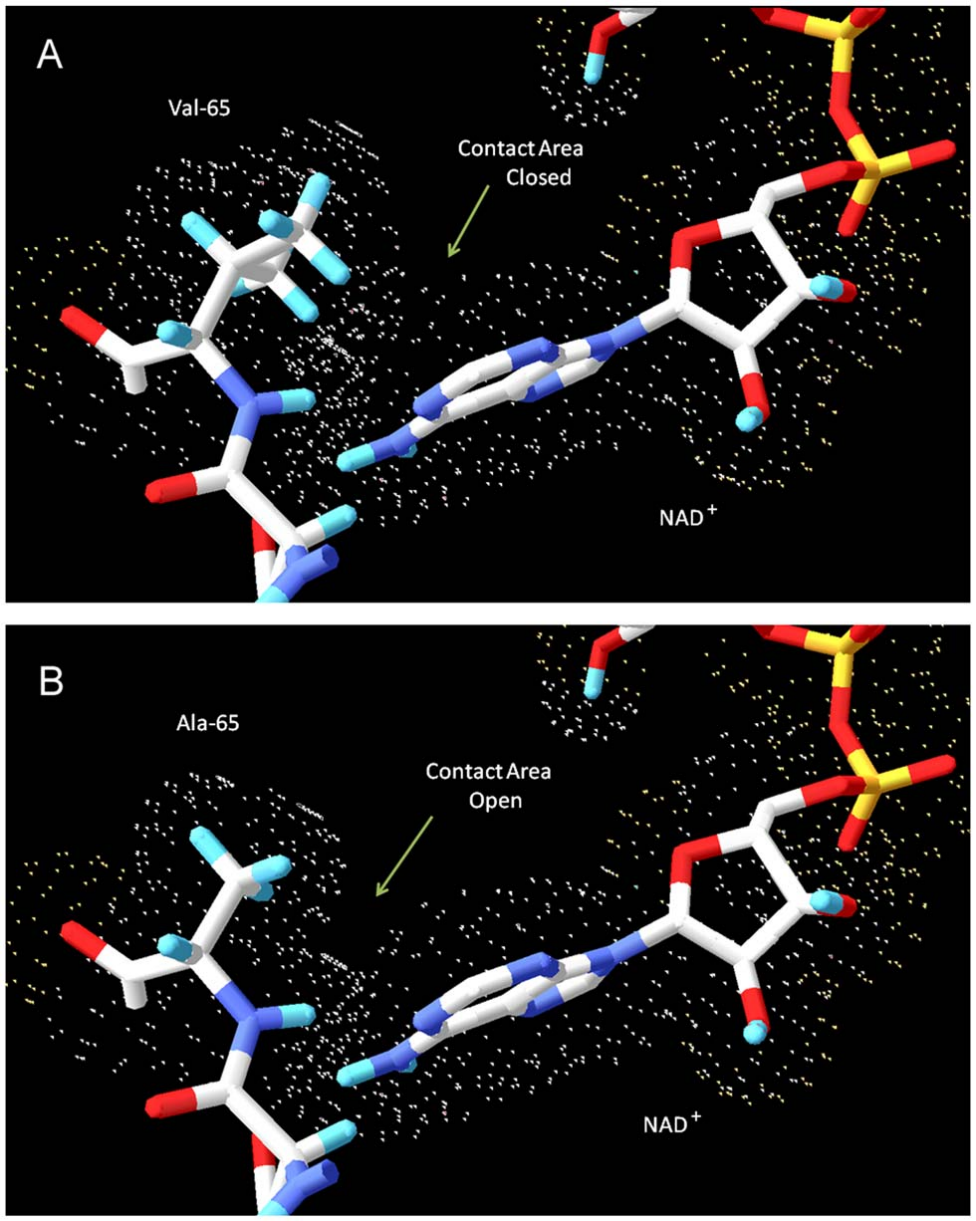
Van der Waals interactions between the adenine ring of NAD^+^ and the side chain of residue 65 of HSD10. The wild type HSD10 and mutant HSD10 were shown in part (A) and (B), respectively. Different colors represent different atoms: carbon (white), hydrogen (blue), nitrogen (purple), phosphorous (yellow) and oxygen (red). For clarity, all other amino acid residues in the protein, other than the neighboring aspartate 64 have been rendered invisible. Small dots represent the extent of the van der Waals radii for atoms in amino acid residue 65 in the protein and in the NAD^+^.

**Figure 5:**
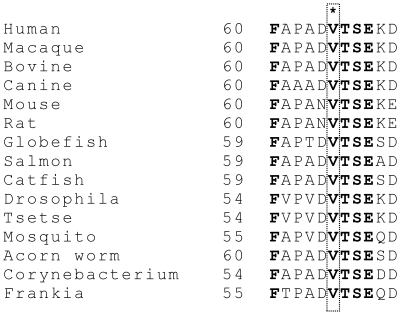
Comparison of amino acid sequence around valine 65 of HSD10 with those of its othologs in different species. Residues conserved in all species were bolded. The asterisk * indicates an extremely conserved residue of this NAD^+^-dependent dehydrogenase.

A novel mutation c.194T>C transition was identified in the *HSD17B10* gene of the patient (Fig. 2). In contrast, there is no mutation found in the same gene of his sister. RFLP analysis revealed that the patient's mother is an asymptomatic carrier, and also confirm that the patient's sister is homozygous for wild-type *HSD17B10* (Fig. 3). A genetic pedigree of this family is shown in the Fig. 3A. The patient suffers from an X-linked intellectual disability although he is the only affected individual in this family. Most female carriers are asymptomatic because of heterozygosity in their two X chromosomes (M1–6 of Fig. 3B) and because the mosaics of the *HSD17B10* gene expression [15]. This novel c.194T>C transition in the *HSD17B10* gene results in a substitution of alanine for valine at residue 65, which is located in the active site of HSD10. This valine residue interacts with the adenine ring of the coenzyme NAD^+^ (Fig. 4). Valine 65 is extremely conserved in HSD10 and its othologs in various species from mammals, fishes, insects to bacteria (Fig. 5).

## Discussion

The pathogenetic mechanism of HSD10 deficiency remains largely unknown. HSD10 deficiency was originally described as a defect in isoleucine metabolism, although attempts at treatment with isoleucine and protein restriction did not clearly alter or improve the course of the disease despite reduction in the excretion of 2-methyl-3-hydroxybutyrate and tiglylglycine [11]–[14]. With the finding that a silent mutation in *HSD17B10* (previously denoted *HADH2*) [16] was associated with normal isoleucine metabolism in a family with nonprogressive, syndromic form of X-linked mental retardation (MRXS10) (OMIM#300220), the possibility of other disease mechanisms was suggested [2], [17]. MRXS10 was described in a single family with mild mental retardation syndrome associated with choreoathetosis and behavioral disturbance with normal carrier females [18]. Imbalance in neurosteroid metabolism has also been suggested as a pathogenic mechanism of *HSD17B10* mutations [8].

Compared with previously reported HSD10 deficiency patients, whose *HSD17B10* gene bears a c.388C>T variant [8]–[10], the current patient has much milder neurological symptoms that are somewhat similar to patients with MRXS10 caused by a silent mutation in the *HSD17B10* gene [17], [18]. Available data have not yet convincingly demonstrated that the levels of HSD10 protein or activity are substantially reduced in lymphoblastoid cells from MRXS10 patients [2]. Although it remains to be seen how a mutation in the *HSD17B10* gene or a deficiency of the HSD10 enzyme leads to any pathology, some have pointed to pathologies arising from defects in neurosteroid metabolism [19] or in the formation of protein complex as the core of mitochondrial RNase P [20]. Our recent study supports the theory that a pathological imbalance in neurosteroid metabolism could be a major cause of the neurological abnormalities associated with HSD10 deficiency [8].

**Figure 6:**
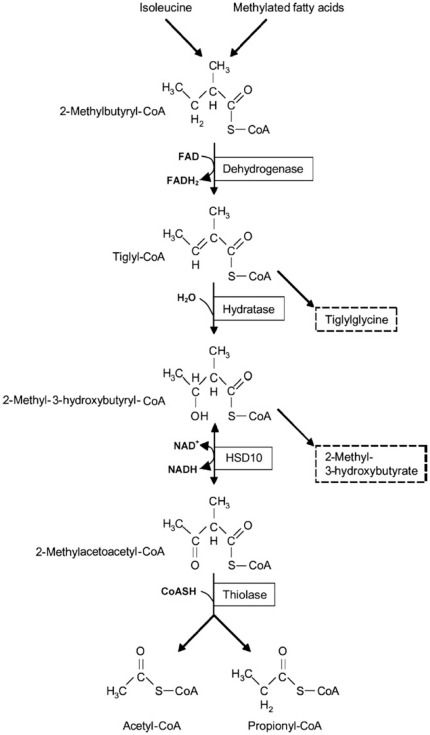
Isoleucine and methylated fatty acid oxidation pathway. Compounds in dashed line boxes were increasingly excreted from patients with HSD10 deficiency or beta-ketothiolase deficiency (adapted from Ref. 2).

Significantly elevated levels of 2-methyl-3-hydroxybutyric acid and tiglylglycine in the patient's urine (Table 1) unequivocally indicated that there is a block in isoleucine degradation at the fourth or fifth step of this catabolic pathway (Fig. 6). A diagnosis of β-ketothiolase deficiency (OMIM#203750) was not supported in this patient based on the following observations: (1) the patient has no history of metabolic acidosis, (2) the patient's HSD10 rather than β-ketothiolase activity was much lower than the normal control level, and (3) no 2-methylacetoacetic acid was detected in his organic acid profile. The accumulation of isoleucine metabolites in this patient points to HSD10 deficiency (OMIM#300438) instead. HSD10 catalyzes the fourth reaction of the isoleucine catabolic pathway (Fig. 6) and was first isolated by Schulz group [21]. The human *HSD17B10* gene, encoding HSD10, formerly also known as short chain 3-hydroxyacyl-CoA dehydrogenase (SCHAD) [22], was then cloned and mapped to Xp2.11 by Yang and co-workers [23]. It is well established that HSD10 deficiency, formerly MHBD deficiency, resulted from a missense mutation in the *HSD17B10* gene [8]–[10]. Identification of a missense mutation in the *HSD17B10* gene of the patient and his mother is necessary for diagnosing HSD10 deficiency, an X-linked intellectual disability (Figs. 2 and 3). Since the reference DNA sequence of the *HSD17B10* and its surrounding genes had been corroborated in more than 2,500 X chromosomes of normal control subjects [17] and the variant detected in this study was not found in the YH Genome of the Beijing Genome Institute (http://yh.genomics.org.cn) and the database of JSNP (http://snp.ims.u-tokyo.ac.jp), c.194T>C is a clinically associated mutation rather than a single nucleotide polymorphism of the *HSD17B10* gene in the Far East population. The highly conserved valine residue (Fig. 5) is replaced by alanine in the mutant HSD10. Since the side chain of valine 65 is so close to the adenine ring of the co-enzyme, the missense mutation p.V65A results in a loss of two forked methyl groups that weakens interactions between residue 65 of HSD10 and NAD^+^ (Fig. 4).

It was observed that the HSD10 activity in the patient's cells is only about half of that in normal controls under experimental conditions of this study. The reduction of HSD10 activity due to the mutation may account for the accumulation of isoleucine metabolites in the patient's blood, reflected in increased excretion in the urine, while his beta-ketothiolase (β-KT) activity was found to be almost in the normal level. Of course, damage to this enzyme would affect not only its catalytic versatility but also its non-enzymatic functions, e.g., its binding capacities as reviewed recently [24]. To elucidate the pathogenic mechanism of HSD10 deficiency, much more research needs to be done in the future.

Finally, although previously reported HSD10 deficiency cases who were either of Spanish or German descent, the results of this study demonstrate that HSD10 deficiency may affect other ethnicities.

## Materials and Methods

### Ethics Statement

This study has been approved by the Internal Review Board of NYS Institute for Basic Research in Developmental Disabilities, and the written Consent for Publication was acquired from the patient's parent.

### Gene Cloning and Sequencing

Human chromosome DNA was isolated from 100 µl of blood by use of the DNeasy Blood & Tissue kit (QIAGEN, Valencia, CA) according to instructions of the manufacturer. The *HSD17B10* gene at X chromosome was amplified with a pair of primers, HSDF and HSDR (Table 1S of [8]), by PCR (94°C 3 min; 35 cycles: 94°C 15 s, 68°C 4 min; 68°C 4 min). A 3.7 kb DNA fragment was purified from the PCR product using the QIAquick Gel Extraction kit according to instructions of the manufacturer. After the PCR product was cloned into the pGEM-T Easy vector (Promega, Madison, WI), JM109 High Efficiency Competent cells were transformed by the pGEM-T Easy- HSD17B10 according to instructions of the manufacturer. Amplified pGEM-T Easy- HSD17B10 was isolated from transformants employing the QIAprep Spin Miniprep kit, and then used as the DNA sequencing template. The DNA nucleotide sequence was determined by the dideoxy method with ten different sequencing primers [8]. Raw DNA sequence data from the DNA Analysis Facility at Yale University were aligned and analyzed with the SDSC Biology WorkBench Version 3.2, and compared with the normal X chromosome DNA sequence (accession #Z97054) to identify a mutation(s) present in the *HSD17B10* gene.

### Restriction Fragment Length Polymorphism (RFLP) Analysis

The pGEM-T Easy-HSD17B10 isolated from 18 different colonies of the transformants was digested as 18 individual samples by *BstE*II at 60°C for 1 h, and then the restriction products from each sample were separated at 1% agarose gel.

### Protein and HSD10 Assay

Protein concentrations were determined by use of the Micro BCA protein assay kit (Pierce, Rockland, IL) according to the instruction of the manufacturer. Measurements of HSD10 activity in lymphoblastoid cells were performed as described previously [12].

### Quantification of Urine Organic Acids

The organic acid profiles were determined by gas chromatography-mass spectrometry [25].

### Analysis of the three dimensional structural relationship of Residue 65 of HSD10 and NAD^+^

The crystal structure of human HSD10 complexed with NAD^+^ was used as the template structure [26]. The 1U7T.pdb file was obtained from the Protein Data Bank (www.resb.org) and visualized using DeepView/Swiss-pdb Viewer 3.7 [27]. Hydrogen atomes were added to the X-ray-derived pdb file using What If [28].
